# A Practical Essay on Some of the Principal Surgical Diseases of India

**Published:** 1841-10

**Authors:** 


### Art. VII.

*A Practical Essay on some of the principal Surgical Diseases of India.* By F. H. Brett, Esq., Bengal Medical Service, Surgeon to the Governor-General's Body-Guard.—*Calcutta*, 1840. 8vo, pp. 506. Plates.

There are obvious reasons why the work before us should be treated tenderly: it is the first important surgical contribution from our brethren in India; it has been written in India by an Indian surgeon; it has been printed in India, and printed not by professed workmen but by amateurs. Still, as we have ever regarded the system of indiscriminate praise adopted (from what motives we shall not enquire) by some medical reviewers, as alike degrading to the author and the critic, we shall not deviate from our honest and independent course in the present instance; and though we shall sincerely regret if we cannot commend Mr. Brett so highly as we would desire, he will, at least, have the satisfaction of knowing that when praise is bestowed, it is bestowed deliberately and from conviction. High praise he unquestionably merits for having written the book at all; nor can any imperfections it may present of plan or execution deprive him of this. There is so much to be done to make us acquainted with the modifications which difference of country brings about in the great features of disease, that we must foster every instalment we get, even although it do not come up to a standard we may ourselves frame.

We commence, then, by saying that we have been rather disappointed by Mr. Brett's book. It will certainly do much less for surgery than has been done by Annesley, Twining, and others for medicine in our Indian empire. We do not, however, by any means wish it to be understood that Mr. Brett's work, as far as it goes, is not the work of a sensible and well-informed and experienced surgeon; what we complain of is, that it is more "Surgery in England" than "Surgery in India." What we expected and wished for was not a compilation, however well it might be done, but an account of the changes produced in diseases common to both countries by the climate of India; and also to know how far it modified operative surgery. In this respect we are bound to say it has in nowise satisfied our expectations.

The volume is divided into seven chapters which are devoted to the following subjects: Inflammation, and its Sequelæ; Parasitic Growths; Indian Leprosy; Diseases of the Generative and Urinary Organs; Diseases of the Eye; Autoplastic Operations; and the Dracunculus: a list which necessarily leaves many important surgical subjects unnoticed. We see no better mode of giving our readers an idea of the contents of this work than by offering any remark which may occur to us on each chapter in the order in which they stand.

The subject of Inflammation has been so lately discussed in our pages that we will not enter upon the opinions of Alison and others which form the staple of our author's observations in chap. i.; but connected with the subject are some excellent observations on the health of Europeans in India, which, on account of their practical value, we here extract:

"The inflammatory diseases of Europeans, in India especially, are chiefly induced by the highly stimulating diet they use and the little exercise they take, aided by the debilitating circumstances to which they are exposed in this climate; rendering them more liable to the exciting cause of fever, viz. malarious atmosphere. A European on his arrival in India is generally in the highest state of plethora. By such a system of stimulating diet he seldom escapes a severe attack of inflammation shortly after his arrival. .......... On convalescence the same habits are renewed, and he arrives in the upper provinces with his health restored. For the first few years of his residence in India he becomes in high condition, especially during the cold season.......But his system both vascular and nervous is under a constant state of excitement from the continued in-pouring of nutriments, stimulation, and high temperature, and he labours under plethora from *repletion* as well as plethora from *diminished secretion.* He is on the precipice of disease, and a slight impulse is sufficient to destroy the balance and precipitate him. Should he survive the repeated attacks to which he is liable his constitution becomes enfeebled, his appetite impaired, his energies diminished, the secretions and excretions scanty, and the skin dark, harsh, and dry. He is perhaps dyspeptic, and, in a word, his system becomes lowered down to the climate, and he is no longer recognizable as the energetic active European. His muscular system is much reduced. He is the 'dried-up Indian,' with habits and constitution scarcely adapted for his native land. On the other hand, should his constitution still remain of the plethoric tendency, he is gross, corpulent, and flabby. .......... If Europeans cannot adapt their mode of life to a tropical climate, but must indulge in habits totally incompatible with such a climate, they should at all events endeavour to convert their food into wholesome nutriment, and preserve the robustness of their frames by practising athletic exercises in the cool of the day, and wrestling in imitation of the Puh,lwans. It is indisputable that these individuals enjoy an immunity from disease unknown by others. There are none whose constitutions resist the exciting causes of disease so well, although their blood is abundant and their vascular system vigorous. The few Europeans who have entered thoroughly into the spirit of these exercises return to their native land with vigorous constitutions capable of really enjoying their native country. The writer has long admired and practised the calisthenic exercising of the Asiatics, and attributes a better state of health and stamina, and a capability for active pursuit, far superior to that enjoyed by him in England, to a systematic use of these exercises." (pp. 39-41-2.)

In treating of Parasitic Growths some interesting remarks are made upon diseases which happily are unfrequently met with in Europe, viz. Hypertrophy of the scrotum and pudenda; and yet there is little in which the author has not been anticipated, by many years, by Delpech; even his "improvements in the operation" for their removal do not seem new to us. Still India affords a larger field for the observation of those affections than Europe, and it is well to have the accuracy of Delpech's views on the subject confirmed. In 1837 our author removed seven of these deformities weighing in the aggregate, when divested of their fluid, 280 pounds. The results of those operations were favorable; five of the patients recovered, two died of gangrene of the parts, which the author attributes to the operation having been performed during most sultry weather, in a Bengal climate, and in a very crowded hospital, where something like hospital gangrene prevailed at the time. It does not appear that in India the disease falls with greater severity on the sedentary than the laborious portion of the population, though such an opinion has been more than once expressed. Whether the disease be what our author supposes it to be a pure vascular sarcoma, is more than questionable. If he be correct in the opinion that "it is evidently the same disease with that which occurs in the West Indies, Africa, Ceylon, and occasionally in Europe," it is certain that his impression as to its nature is incorrect, unless he have some new view of what constitutes vascular sarcoma. Certain it is that the disease in the West Indies and in the south of Europe is an hypertrophy of existing tissues, with serous infiltration; whether dependent on lymphatic disease may be doubtful.

The section on Bronchocele is interesting; but we entered so fully on this subject in a previous Number (vol. VIII. p. 103), that we must pass it over here.

On Elephantiasis or, as our author prefers to call it, Lepra Tuberculata Virulenta, there is an interesting chapter. The symptoms of the disease are as follows:

"An individual labouring under this complaint usually presents himself in the following condition: A general torpor seems to pervade his system, so that his sensations of pleasure and pain are considerably impaired. Instead of that excessive propensity to venery which they are generally supposed to possess, they have usually little or no inclination for such indulgence, though there are exceptions to this, where it exists in a diminished degree only. The pulse is slow, not small, but heavy, 'as moving through mud.' There seems to be a sort of stagnation in the process of nutritive vitality. The bowels are generally constipated. The patient's face is bloated. His forehead, nose, lips, and ears become swollen; his nostrils expand; his eyes appear sunk and fiery, the tone of his voice is altered to a loud and nasal sound; the skin, especially of the extremities, is harsh and scaly, resembling a case of ichthyosis. He is subject to profuse perspirations, especially when exposed to the sun; these are confined to the trunk, not the slightest moisture permeating the surface of the scaly extremities. He is often distressed with thirst and a sensation of internal heat. The knees are stiff, and their motions contracted. The hairs generally fall from the brows, the beard, the pubes, and axillæ, &c. or they are seen stunted and dried up for want of moisture. His breath is fetid, and his perspiration rank and offensive. The blood drawn by venesection is very dark. A kind of dry gangrene pervades the fingers and toes, which are generally eaten away, and drop off at the first phalanges; these sores then generally cicatrize over without any rete mucosum, and the next joint becomes invaded by a renewal of the ulcerative process. It is singular to observe that notwithstanding these extensive sores, they can wear hard shoes, which are seen saturated with the sanious ichor that exudes from these ulcerated surfaces. The disease progressively advances, eating through the ankles and wrists, and performing slow but certain and successive dismemberments, every revolving year bearing some trophy of this tardy but gradual march of death, till at last the vitals become affected. During all this, a sleepy inertness overpowers his mind, and 'seems to benumb and almost annihilate every faculty, as well of the soul as of the body, leaving only sufficient sense and activity to crawl through the routine of existence.' In the last stages of the complaint, the flesh gapes with long sores, the mouth, nose, and brain become exposed to its ravages, and death at length terminates this loathsome existence. He is usually cut off by the supervention of diarrhœa. It is astonishing, however, to witness how long the victim lingers, from twelve to twenty years being no uncommon duration. During the greater part of this period he has a good and even voracious appetite, and moves about from village to village." (pp. 163-5.)

The causes of the disease are not very evident though the inclination of opinion favours the belief that improper food is a principal element in its production. An analogy is thought to exist between this disease and that from which Europeans have suffered from the use of spurred rye; and it is strengthened by the evidence that it has prevailed to the greatest extent in those districts and seasons in which the poorer classes were obliged to eat diseased rice. "The disease is very common in the valley of the D,hoon and in Bengal, in both which provinces coarse rice constitutes the ordinary article of diet."

Our author gives evidence in favour of the non-contagious character of the disease; but much well-considered evidence will be necessary to weigh down the mass of facts which have been collected to support an opposite opinion. Certainly the faith in its contagion is waning among the natives; for in the present day we no longer hear of lepers being buried alive, drowned in the Ganges, or immolated on the Suttee; though it is said they occasionally commit suicide by leaping into the Ganges.

In the early stages the disease is susceptible of cure, though even then the failures are frequent; in its advanced stages much relief may be afforded to the sufferer, but rarely is a patient cured. Arsenic is the remedy most generally employed, but it is associated with the mudārr (Asclepias gigantea) to which Playfair, Robinson, and others have ascribed great virtue. The most accredited form is the following: Oxide of arsenic, 55 grains; mudārr powder, 4 ounces and 80 grains; black pepper, 9 ounces: the whole well beaten at intervals for four days, made into a mass with water and divided into 800 pills. Not more than two pills should be taken daily, which are equal to about a seventh of a grain of arsenic. Mr. Marshall treated 200 cases with nitric acid, a drachm in a pint or a pint and a half of water daily; above one third were cured and the remainder were greatly benefited. The Hindoo physicians place their patients during treatment on the following diet: all kinds of game, Sushtiká rice, and Urhur.

Urinary Calculi are of frequent occurrence in India, and happily the operation for their extraction seems to be attended with less risk than in the continent of Europe; whether this is to be attributed to the use of vegetable food impressing a difference of constitution upon the natives of India, or whether it be owing to the temperature of the climate, is a point of great interest, but which we want the elements to determine. In our own country we want the data to determine whether our agricultural peasantry, consuming as they do (more especially in Scotland and Ireland), comparatively very little animal food, do better after great operations than those whose dinner is daily composed of meat. Our county hospitals should assist in the determination of this question. Again the question of temperature in its influence upon wounds is of vast importance to the surgeon. The evidence of Larrey, of the rapidity with which wounds healed during the occupation of Egypt by the French army, the experiments made by Breschet and others, the use of warm-water dressings in our own country, favour the idea that a comparatively elevated temperature is favorable to the healing of wounds. If this article should fall into the hands of Mr. Brett we would venture to suggest to him the value of a register for the purpose of ascertaining the ordinary period required for the cicatrization of amputated surfaces in India.

To return to calculous diseases. Some persons have attributed the frequency of stone in India to the errors of diet in the natives, especially the children, who are permitted to devour various kinds of unwholesome rancid sweetmeats, and particularly coarse unleavened bread, at all hours of the day. As an evidence of the frequency of stone cases, our author states that the late Mr. Burnard cut about forty patients for stone at the city of Benares; that he himself has operated on four patients in one day, all residing in the same village or its immediate neighbourhood; and that he had attended upwards of 100 cases during his abode in India. In "the natives of India," says Mr. Brett, "their systems are habitually prepared for operation, and the little advantage derivable from preparatory treatment is more than counterbalanced by fear and suspense. They come with confidence in the superiority of European skill, from hearing of our success on other sufferers, and they seek for speedy relief from agonizing pain. The patient is infinitely more pleased by immediate operation, which when set about with the least possible ceremony, is not looked upon at all seriously by him." This system might be beneficially applied elsewhere than on the continent of India. We are convinced that it might be advantageously followed amongst ourselves. The state of feverish anxiety and irritation, which is frequently developed in the patient during the time of preparation, is often extremely prejudicial to the success of operations. We by no means wish to advocate a system which has been too generally followed by some of our continental brethren, of acting in every case without submitting the patient to any preparatory treatment. In getting rid of one evil, as far as may be prudent, it surely is not necessary to adopt another. There are many cases where such preparation is unnecessary, and where in fact it exercises an injurious influence; there are others in which it cannot be dispensed with. Mr. Brett has, of course, made up his mind as to what constitutes the state of habitual preparation for operation which characterizes the natives of India; and we hope some day to be favoured with his views on the subject.

Mr. Brett in all cases causes to be administered to his patient an hour before the operation, not less than 100 drops of laudanum; and as this is the only feature, as connected with the operation, differing from those ordinarily employed, it would be interesting to know how far this has contributed to his brilliant success. His last sixty-eight cases all did well, although operated upon in different districts, viz. Calcutta, Cawnpore, Meerut, Shahjehanpore, Delhi, and Mooradabad, and although six of the patients were sixty years of age. Malgaigne has for some years strongly recommended the use of large doses of opium, persisted in at short intervals, so as to keep down all nervous excitement, and he has adduced a considerable mass of evidence in support of the success of such practice. The natives have a method of fomenting the abdomen after the operation, which, associated with castor oil, is believed to relieve a condition simulating peritonitis, with nausea, tumefaction, and constipation, which occasionally comes on from twenty-four to thirty-six hours after the operation. "A square piece of cloth of a foot diameter is dipped in oil and placed on the abdomen. Small cloth bags, containing equal parts of potash and common salt heated on an iron plate, and placed on the fire, are made excessively hot, and alternately applied to the oiled cloth, keeping up an incessant steaming heat." In our own country similar though perhaps less energetic means for the same purpose, in the shape of hot bran, oats, or salt in bags, or turpentine fomentations, or hot poultices, are employed, and their efficacy under such circumstances is generally admitted.

Our author seems but little acquainted with Lithotrity, either practically or historically; and considering how successfully he performs cystotomy perhaps it might be as well he should continue so. He says "lithotrity can only be applied to stones of small size, not more than an inch and a half in diameter." How the *lithotriteurs* and *percuteurs* of the present day would smile at the idea that their empire was to be circumscribed within such narrow limit!

In treating of Gonorrhœa, Mr. Brett gives an explanation of the mode in which chordee is produced, which we strongly suspect presents more of novelty than reality: "A very distressing and painful symptom is liable in severe cases to occur, especially during the night, termed chordee. The inflamed membrane is stretched, and great pain is experienced along the course of the urethra. The cause of this is the inflammation having taken place in one or more of the large lacunæ, extending to the cellular tissue and substance of the corpus spongiosum urethræ or the corpus cavernosum; and according as one or other of these structures is affected, so is the penis bent down or towards one side." Now, what proof can he give us that inflamed lacunæ are the cause of chordee? Is it not quite as philosophical to assume that inflammation has extended from any other portion of the mucous membrane to the adjacent structures as from the lacunæ?

Again, he says in an equally off-hand manner, "It has been well ascertained that females not labouring under virulent gonorrhœa themselves are yet capable of imparting the disease, especially to irritable habits." Does Mr. Brett mean that gonorrhœa is not always the consequence of a virus, or does he suppose a virus is not specific in its character? Does he suppose that vaccine virus or pus from any other source placed upon the mucous membrane of the urethra would develope a gonorrhœa virulenta, as he is pleased to term it?

In speaking of one of the consequences of gonorrhœa, Stricture, he says, the most usual seat of stricture is six and a half or seven inches from the external orifice. Now we will undertake to say that not one case in a hundred is seated so far back in the urethra as seven inches or even six and a half from the orifice. We would ask the author how many times he has seen the bulb six inches from the orifice. The curvature is the ordinary seat of the disease, and in an overwhelming majority of cases the curvature is not more than five inches and a half from the orifice.

Our author, when speaking of Extirpation of the Testicle, says, "should the spermatic cord have retracted into the abdomen it will be necessary for the surgeon to slit up the abdominal ring and pull down the artery by means of a hook." This is a caution commonly given and no doubt a proper one, but we may venture to ask whether he ever knew such an accident to occur? We ourselves happen to know three cases, where, under particular circumstances, the scrotum and testicles have been shaved off with a razor, and where there was not much hemorrhage or any retraction of the cord into the abdomen.

With great respect for Mr. Brett's opinion, we do not think it by any means proved that Cirsocele is most liable to occur on the left side, *because* of the pressure of the sigmoid flexure of the colon, commonly full of fæces, on the spermatic vein at the angle at which it enters the ascending vena cava. We are quite aware that Pouteau threw out the idea long ago, but he only suggested that it might be an occasional cause. Has Mr. Brett found after death the sigmoid flexure full of fæces? Has he distinguished it among the living? Does he know how many young men presented as recruits or conscripts exhibited this condition? Does he think the explanation accounts for the greater frequency of varicosed veins in the left leg than the right?

We should not like to submit our patients to the treatment he suggests for varicose veins of the scrotum and cord:

"In obstinate cases obliteration of the veins may be accomplished by the application of a heated wire; for which purpose the upper part of the tumour should be grasped and made prominent, when a red-hot needle may be pushed through the veins at several points. The patient should be made to stand before the surgeon, who then examines the component parts of the cord; he should then return to the horizontal posture, when the turgescence of the veins will subside, and the vas deferens be distinctly felt; this is to be held by the finger and thumb of the operator's left hand while the patient rises, and again the veins become turgid, which should now be pierced with the cautery at a black heat in one or more points." (p. 299.)

The chapter on Diseases of the Eye is composed principally of selections from Mackenzie, Lawrence, Travers, and Morgan, and contains little, probably, with which our readers are not familiar.

We could have wished that some attempt had been made to clear up the early history of *autoplastic surgery* in India. In connexion with the subject a curious fact is mentioned by our author, which, as he calls it so, we are bound to receive as an established fact:

"An interesting fact, which may be turned to much practical advantage, has been well illustrated in India, viz. that mucous membrane not only becomes integument when everted and exposed to the atmosphere, but that it becomes *black* by the deposition of pigmentum, and that integument when inverted becomes mucous membrane, as is exhibited in the enormous tumours of the scrotum where the extended prepuce becomes mucous membrane." (p. 454.)

That accidental mucous surfaces may result from the modification of cellular and other tissues is an admitted fact in science; still no one maintains that an accidental mucous surface ever acquires the exact organization of proper mucous tissues. As to the conversion of mucous into proper cutaneous tissues we have never seen anything to prove it, and this not from want of opportunity. We have all seen cases of old prolapsed uterus and rectum; we have seen that the mucous surface has become thick, dry, and coriaceous, but still having little in common with the cutaneous tissues, and certainly we have never in any such cases been conscious of the pigmentary deposit to which our author refers; still the thing may happen in India.

The last chapter treats of the Filaria Medinensis Dracunculus, or as it is commonly known in this country, the Guinea Worm. Although the opportunity of seeing the animal in our own country does not often occur, yet it is occasionally presented in sailors who trade to Guinea and the adjacent coast, and as the subject is one of considerable interest, and our author's opportunities of observing it have been frequent, we will profit by his experience on the subject. He says that worms resembling the filaria are found in the waters of the D,hoon where also the disease is extremely prevalent; he refers to Chisholm and Morehead, who think that the localities in which dracunculus prevails are in districts the rocks of which are of the secondary trap series; but it appears that the D,hoon derives all its waters from the Himalayan Chain, and that so far the secondary trap series have nothing to do with the prevalence of the dracunculus. As evidence against the opinion some time entertained by Mylne and others, that the dracunculus was only a diseased absorbent, he refers to the testimony of many medical officers who had seen the worm exhibit every sign of life after its extrication from the body. Dr. Smyttan has found the same animal attached to the peritoneal covering of the liver and to that of the left kidney, which was writhing alive, and partly floating among the viscera. What our author considers to favour the idea that this worm may be introduced from without and not generated in the system, is that no case of dracunculus occurred in the Governor-General's Body-Guard during the first year's occupation of the valley. All occurred on the second year's cantonment at Deyrah and during the following years on the regiment's return to Calcutta, and that the troopers of the body-guard when quartered at Deyrah obtained their water from a mountain stream particularly reputed for purity.

"It is probable that the young when once imbibed into the system, and when not larger than a minute hair, threads its way through some capillary pore or secreting orifice, and finds its exit into the cellular tissue, where it derives its nourishment and grows, and as it migrates and increases often excites a real diffuse phlegmonous inflammation. A formication commences under the skin accompanied with a superficial cord-like elevation on the surface. A phlysacious vesicle or pustule forms which bursting gives exit at a circular aperture either immediately or after suppurating for a day or two to the head of the worm. These local symptoms are preceded usually by a slight derangement of the system generally. When situated about the fingers or toes, the worm is often productive of much suffering, and is with difficulty got rid of. When deeply seated it sometimes causes considerable fever, great swelling, and tedious abscesses and sinuses, giving out a serous ill-conditioned discharge for months after making its appearance." (p. 479.)

"When the animal can be distinctly felt coiled under the skin, an incision may be made by the side of it, and a portion of the worm seized and gently pulled forth. The natives are exceedingly careful not to wound it, and attribute the severe suppurative inflammation to any broken portion of the worm remaining. . . . . Certain it is that very severe abscesses form after the breaking off a portion of the worm, indeed the natives generally come to us when the mischief has occurred from their taking out the worm at their own homes. A free incision having been made over the worm, it is to be seized and gently brought forth by means of a bent probe. Should the animal's head have presented through the phlysacious pustule it may be carefully pulled forth and turned round a small rolled piece of adhesive plaster. I have succeeded in this manner in rolling off three feet of the worm in the course of an hour. This may be favoured by gentle pressure on the skin over the worm, and by pouring a stream of cold water over the part." (p. 487.)

We regret that our present space does not allow us to make further extracts from Mr. Brett's volume. Although, as we have said, not exactly according to our notions of what such a work should be, it will be highly useful to Indian surgeons, and still more so to European surgeons proceeding to India. No one who reads it can doubt that its author is a most zealous and accomplished surgeon.

---

